# P307: Accidents involving exposure to biological material, post-accident conducts and immunization coverage between manicures/pedicures in Brazil

**DOI:** 10.1186/2047-2994-2-S1-P307

**Published:** 2013-06-20

**Authors:** JL Garbaccio, AC Oliveira, AO de Paula

**Affiliations:** 1 Nursing, Federal University of Minas Gerais, Belo Horizonte, Brazil

## Introduction

The literature indicates that outbreaks of viral hepatitis B may occur with the contaminated materials from manicure and pedicure. In Brazil, there is an important concern due to the habit of removing the nail cuticles that causes bleeding.

## Objectives

This study aimed to identify the occurrence of accidents involving exposure to biological material, immediate actions after accidents and vaccination coverage in these professionals.

## Methods

A Survey was run with a random sample of 200 professionals, older than 18, covering 200 beauty salons in Belo Horizonte, Brazil. A questionnaire was used (Jul/12-Jan/13) to obtain information of demographics, accidents involving exposure to biological material, immediate actions after accidents and vaccination coverage of these professionals. The results were analyzed by the software SPSS. The Ethics Committee of the Federal University approved the study.

## Results

All professionals interviewed were women, the average age was 30, have 10 years of experience (11%), work an average of 8 hours per day (57%), employed less than a year in that salon (34%). About education 55% had completed high school, 54% had done some training course in the field, yet 38% became manicure/pedicure by their own initiative. Moreover 76% hadn’t got instruction about biosafety. Among the 200 participants, 66% reported having experienced accidents with needlestick material working. There was no statistical association (p>0.05) between demographic profile and accidents, except for education level. Those with complete high school qualification have injured the most (38%/p=0.02). In 65% of the cases, pliers to remove cuticle were the agent of the accident. Some professionals (57%) take care of their own nails using the material that was used in their customers and 52% mentioned have been injured while removing their own cuticle. Only 33% washed the local with water after accidents. Regarding immunization status, 54% had received the hepatitis B vaccine and 68% tetanus.

## Conclusion

The results of this research reinforce the biological risk the professionals are exposed to, especially due to needlestick accidents, lack of vaccination and prophylactic actions after the exposure, which minimize the transmission of pathogens.

## Funding Agent

Fapemig PPM nº 00340-11

## Disclosure of interest

None declared

